# Fiber optic backscatter spectroscopic sensor to monitor enamel demineralization and remineralization *in vitro*

**DOI:** 10.4103/0972-0707.44053

**Published:** 2008

**Authors:** Anil Kishen, Annie Shrestha, Adeela Rafique

**Affiliations:** Department of Restorative Dentistry, Faculty of Dentistry, National University of Singapore, Republic of Singapore, Singapore

**Keywords:** Fiber optic, demineralization, dentine, remineralization

## Abstract

In this study, a Fiber Optic Backscatter Spectroscopic Sensor (FOBSS) is used to monitor demineralization and remineralization induced changes in the enamel. A bifurcated fiber optic backscatter probe connected to a visible light source and a high resolution spectrophotometer was used to acquire the backscatter light spectrum from the tooth surface. The experiments were conducted in two parts. In Part 1, experiments were carried out using fiber optic backscatter spectroscopy on (1) sound enamel and dentine sections and (2) sound tooth specimens subjected to demineralization and remineralization. In Part 2, polarization microscopy was conducted to examine the depth of demineralization in tooth specimens. The enamel and dentine specimens from the Part-1 experiments showed distinct backscatter spectra. The spectrum obtained from the enamel-dentine combination and the spectrum generated from the average of the enamel and dentine spectral values were closely similar and showed characteristics of dentine. The experiments in Part 2 showed that demineralization and remineralization processes induced a linear decrease and linear increase in the backscatter light intensity respectively. A negative correlation between the decrease in the backscatter light intensity during demineralization and the depth of demineralization determined using the polarization microscopy was calculated to be *p* = -0.994. This *in vitro* experiment highlights the potential benefit of using FOBSS to detect demineralization and remineralization of enamel.

## INTRODUCTION

Optical diagnosis affords many important advantages over traditional techniques, including the potential to reduce the need for clinical expertise and unnecessary tissue removal, and the potential to reduce health care costs for patients. Many studies in the past have applied different light based approaches to detect incipient dental caries.[1,2] Some of these techniques are now commercially available, while some others are still being evaluated in the laboratories.

Optical properties are an intrinsic property that characterizes biological tissues and tissue fluids. They do not depend on its geometry. In the past, optical properties such as absorption, scattering, and fluorescence were used to characterize biological tissues. Essentially, a fiber optic sensor system can be designed using two optical fibers. Light at a particular wavelength is transmitted through one fiber, while the reflected or scattered or absorbed light is collected and transmitted to a detector through the second fiber. The signal obtained from the detector is proportional to the optical spectrum of the biological tissue. The ends of the fibers are bare and diagnosis is made by direct interaction between light and the tissue sample. These sensors are called direct fiber optic sensors.[3]

In this study, a direct fiber optic sensor system is applied to monitor the demineralization and remineralization induced changes in the backscatter light from the enamel surface. A fiber optic bundle probe is utilized, in which some fibers serve to illuminate the tissue while some fibers serve to acquire the backscattered light from the tissue. A broad spectrum visible light source is used to illuminate the tissue and a high resolution spectrophotometer is used to acquire the complete spectrum of the backscattered light.[3]

The application of visible light fiber optic spectroscopy for biological sensing offers the following advantages:

1. It allows examination of all wavelengths of the spectrum directly from the tissue, in less than a millisecond.
2. It is highly sensitive to minor changes in the optical characteristics of the biological tissues. This method examines all visible wavelengths simultaneously and, therefore, the integration time for data acquisition can be standardized, unlike a scanning system, which would require separate integration per wavelength interval.
3. There are no moving parts in this system, and there is no variability in wavelength between different spectra. This would facilitate calibration transfer between samples and facilitate repeatability between operators.
4. Fiber optic probe enables the spectrophotometer to be located away from the sampling optics, especially if the environment is hostile.
5. The spectrophotometer regularly has multiple channels, and, hence, different parameters can be detected and measured simultaneously. This advantage is handy when simultaneous evaluation of the reference and test samples are to be carried out.
6. The system is lightweight and very easy to get started.[4]

Clinical visual methods of detecting caries of macroscopically intact occlusal surfaces have been shown to have relatively poor sensitivity.[5,6] Monitoring demineralization and remineralization of dental hard tissues is essential for the prevention and minimally invasive treatment of dental caries and hypersensitivity.[7] Light based methods used for the detection of dental caries induced enamel demineralization are conventional radiography,[4] micro radiography,[8] Digital Imaging Fiber Optic Transillumination Imaging (DIFOTI),[8,9] laser induced fluorescence (DIAGNOdent),[10–13] and quantitative light-induced fluorescence (QLF).[14–18] Micro-radiography has been suggested as the ‘gold standard’ in caries detection. It allows the determination of the mineral loss, lesion depth and body of the lesion. However, because of its destructive nature and hazards of ionizing radiation, it cannot be used in clinical situations. DIAGNOdent uses red laser (at 655 nm) induced fluorescence from the enamel surface, to detect early dental caries. This method identifies caries from the increased light-induced fluorescence over the sound tooth.[14] Apparently, the system measures the fluorescence of the bacterial product such as porphyrins within the carious lesion rather than proper crystalline dissolution. This theory is supported by the fact that DIAGNOdent device does not detect *in vitro* lesions produced by acidic buffers, which does not involve microbiota.[16] QLF is based on the ability of enamel to emit yellow light by autofluorescence, when illuminated with blue light from an argon laser or from a xenon-arc source. The presence of caries is found to reduce the intensity of the autofluorescence. In this system, a micro-camera, interfaced to a personal computer, is used to acquire the image, and a software program is used to detect the darker region from the image.[15–18] A DIFOTI system combines fiber optic illumination and digital image processing technique. In DIFOTI, the demineralized region would appear as darker area against more translucent bright background of healthy enamel.[4] This is not a quantitative method and clinicians have to analyze the images in the same way as radiographs.

Earlier experience with the laser-based caries detection system such as QLF demonstrates that deposits like plaque or calculus could give rise to false-positive reading.[19] These systems require improvement for reproducibly capturing images of the suspected area of the tooth surface, determining the depth of the lesion and quantitatively analyzing the detected lesion.[20] In addition, the usage of these systems necessitates a significant learning curve for the clinical examiners. Most of the above disadvantages can be avoided by using fiber optic visible light spectroscopy, which can provide information not only about the intensity change but also the changes in the innate optical spectra of the tissues involved. Moreover, optical measurements with visible light are harmless to both the patient and the operator and fiber optic visible light spectroscopy will provide additional flexibility during instrumentation. Similar fiber optic based system that applied a photodiode as a detector to measure the intensity of the backscattered light has been reported.[21,22] These systems however do not provide the advantages offered by optical spectroscopy.

## MATERIALS AND METHODS

Forty non-carious extracted permanent molar teeth were collected and maintained in deionized water. (It is certified that the collection and use of extracted teeth for this study was approved by the Institutional Review Board of the National University of Singapore.) These tooth specimens were extracted for periodontal problems. The specimens were transilluminated and specimens with cracks, extraction damages, white spot lesions and stains were discarded from the study.

The experiments were conducted in two parts. In Part 1, the experiments were carried out using fiber optic backscatter spectroscopy on (1) enamel and dentine bars (2) sound tooth specimens subjected to demineralization and remineralization. In Part 2, polarization microscopy was used to examine the depth of demineralization in tooth specimens.

### Part 1: Experiments with fiber optic backscatter spectrophotometer

**Figure 1:**
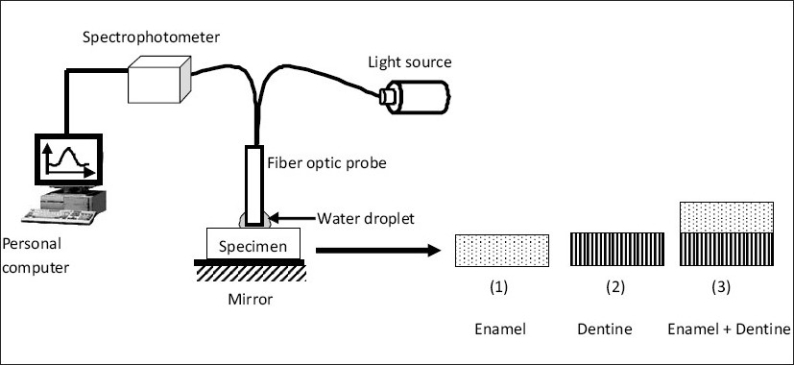
Schematic diagram of the optical sensor arrangement used to test sections of enamel and dentine

**Figure 2:**
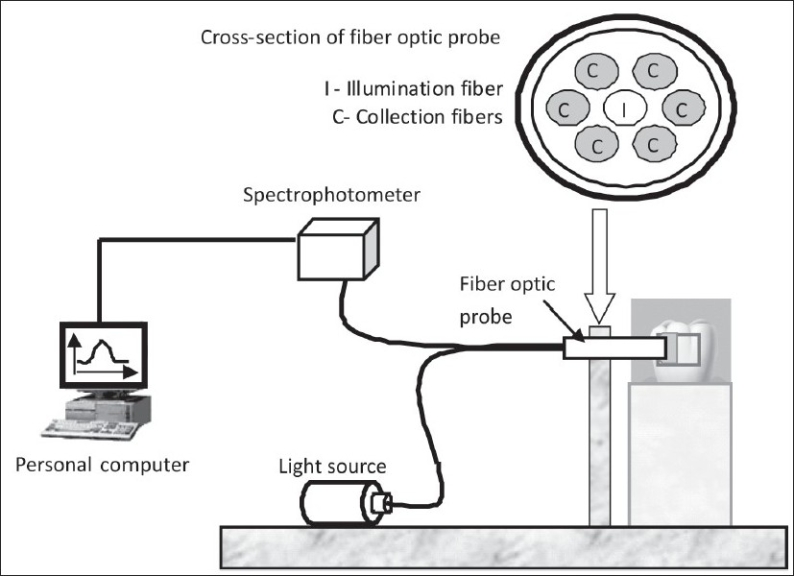
Schematic diagram of the optical sensor arrangement used to test tooth specimens

A reflection/backscatter probe (R400-UV-VIS, Dunedin, Florida, USA) was used for illuminating and collecting light from the specimens. The optical probe consisted of bi-furcated (Y-shaped) fiber assembly, with two fiber bundles of the same diameter, side-by-side in one end (convergent end); they diverged into two arms at the other end (divergent end). The convergent end of the fiber bundle formed the fiber optic probe and was placed on the tooth surface, while the divergent ends were connected to a tungsten halogen light source (HL 2000, Ocean Optics, Dunedin, Florida, USA) and a high resolution spectrophotometer (HR 4000, Ocean Optics, Dunedin, Florida, USA) interfaced to a personal computer. Figures 1 and 2 show the schematic diagram of the optical sensor system used in this study. The fiber optic probe at the convergent end consisted of seven fiber optic cables [Figure 2]. The fiber optic cable in the center illuminated the tooth surface (illumination fiber), while the rest of the six fiber optic cables collected the backscattered light from the tooth surface (collection fibers). The divergent light emerging from the optical fiber was collimated by a spherical mirror in the spectrophotometer. The collimated light was diffracted by a plane grating, and the resulting diffracted light was focused by a second spherical mirror. The spectrum was projected onto a 1-dimensional linear CCD array and the data was transferred to a computer through an analog to digital (A/D) converter (HR 4000, Ocean Optics, Dunedin, Florida, USA). Customized software (OOIBase32, Ocean optics Inc) was used to acquire, store and analyze the spectrum.

The spectrophotometer used had better than 2nm resolutions for wavelength range of 190 to 1100 nm and as fast as 10*µ*s integration time. The spectrophotometer was used to acquire the complete visible light backscatter spectrum from the tooth surface. The integration time in the spectrophotometer is a software controlled variable, which is analogous to the shutter speed of a camera. The integration time specified the amount of incoming photons integrated by the spectrophotometer to generate the given spectrum. The integration time controlled the electronic shutter in the detector, which could be as short as 1 *µ*s, allowing measurement of transient event. It also eliminated light intensity saturation problems.

*Stage 1: Characterization of enamel, dentine and enamel-dentine combination:* In Stage 1, experiments were conducted to characterize the visible light backscatter spectrum from (a) enamel specimens (b) dentine specimens and (c) combination of enamel specimen over the dentine specimen. Four rectangular specimens of enamel and dentine having dimensions of 4 mm (length) × 3 mm (breadth) × 1.5 mm (thick) were prepared and maintained in deionized water. The specimens were tested in three configurations (enamel alone, dentine alone, enamel above dentine) using the FOBSS setup [Figure 1]. A drop of water was placed on the surface of the specimen during measurements, to minimize the effect of surface speckles and to improve coupling of light. The resultant optical spectra were acquired with a constant integration time of 50msec. During data acquisition, three spectra were recorded from each specimen and the spectrum with the maximum intensity was recorded for further analysis. However, obvious difference was not observed between the three spectra recorded from one location.

*Stage 2: Experiments on tooth specimens:* In Stage 2, extracted tooth specimens were tested using the fiber optic spectroscopy system. Twenty four extracted tooth specimens were selected and square windows with dimensions 3mm × 3mm were prepared on the facial and the lingual surfaces of each tooth, using nail varnish. All 24 specimens were soaked in demineralization solution containing 0.05M acetic acid, 2.2mM calcium, and 2.2mM phosphate ions (pH 4.5), to simulate the demineralization process.[23] The specimens were removed from the solution and washed in deionized water for 5 min and the optical spectra from the tooth surface were recorded at 0 (control) and after 24, 48, 72 and 96 hours of demineralization.[24] The schematic diagram of the optical system used for this experiment is shown in Figure 2. After demineralization intervals, the tooth specimens were subjected to remineralization. A remineralization solution containing 0.15M potassium chloride, 1.5mM calcium, and 0.9mM phosphate ions (pH 7.0) was used to simulate the remineralization process.[23] After 144, 168, 192 and 336 hours of remineralization, the specimens were washed in deionized water for 5 min and the optical spectra from the tooth surface were recorded. Throughout the experiment, a drop of water was placed on the surface of the specimen to minimize the effect of surface speckles and to improve coupling of light during measurements. An integration time of 150msec was used to acquire the spectra in stage 2 experiments. The integration time was chosen so that the initial spectra of the control specimen (non-demineralized enamel) were similar to the spectra obtained from the enamel above the dentine configuration tested in the Stage 1 experiment. Each measurement was made three times and the spectrum with the maximum intensity was recorded for further analysis. However, there was no obvious difference between the three spectra recorded from the single location.

### Part 2: Examination using polarization microscopy

In this experiment, twelve extracted tooth specimens were selected and square windows having 3mm (length) × 3mm (width) were prepared on the facial and the lingual surfaces of each tooth, using nail varnish. All these specimens were soaked in the demineralization solution for 24, 48, 72, and 96 hours intervals. After specific intervals of demineralization, three tooth specimens were washed in deionized water for 5 min and stored in plastic containers with 100% humidity before sectioning. Three tooth specimens, not exposed to demineralization, were used as the control sample. A Silverstone-Taylor hard tissue microtome (series 1000 Deluxe, SciFab, Littleton, Co, USA) was then used to obtain several vertical sections of 120±20*µ*m thickness, from each window on the tooth. The tooth sections were initially characterized with an optical microscope and sections damaged during the sectioning procedure were discarded. Water was used as the imbibition solution and the pattern of demineralization in the enamel was determined using a polarized light microscope (Olympus BH-2, Olympus Corp. of America, New Hyde park, NY, USA). The micro Image software (Olympus, Japan), which was interfaced with the microscope, was used to determine the lesion depth.

## RESULTS

### Part 1: Experiments with fiber optic backscatter spectrophotometer

**Figure 3:**
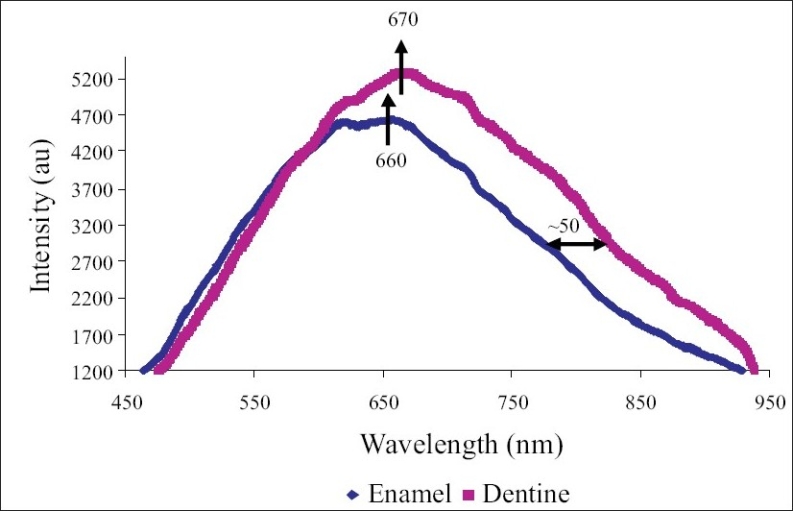
Visible light backscatter spectrum obtained from the sound enamel and dentine sections

**Figure 4:**
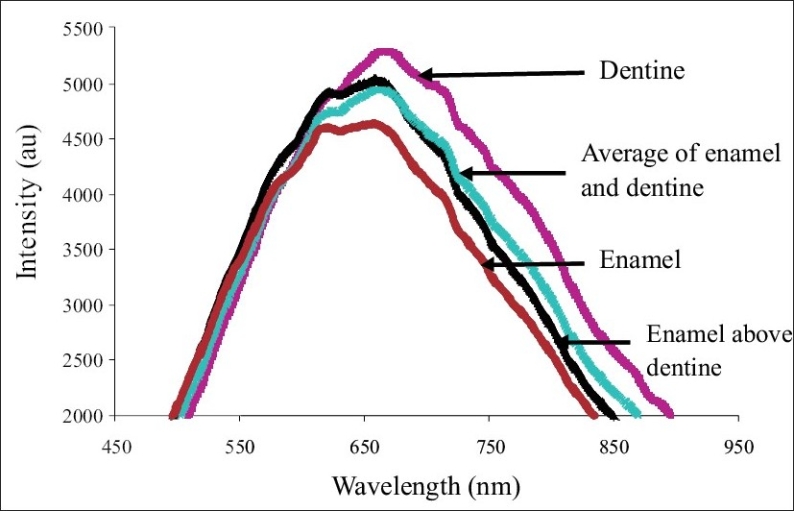
Visible light backscatter spectrum obtained from the enamel, dentine, enamel-dentine combination and the average of the enamel and dentine spectra

*Stage 1: Characterization of enamel, dentine and enamel-dentine combination:* Figure 3 shows the typical normalized backscatter spectrum obtained from the sound enamel and dentine sections. The spectra from all enamel samples had maxima at about 660nm, while the spectrum obtained from the dentine had maxima at about 670nm. The peak of enamel was narrower and flatter, when compared to that of dentine. The width of the dentine peak was about 50nm to the right (towards the infra red region) of the enamel peak, at the half maxima. Figure 4 shows the typical normalized spectrum obtained from the enamel specimen, when placed over the dentine specimen, and the spectrum generated from the average spectral values of the enamel and dentine. A close similarity was observed between the spectra obtained from the enamel-dentine combinations and the spectra plotted from the average spectral values of enamel and dentine. The above spectra resembled more the spectra of dentine than enamel.

**Figure 5:**
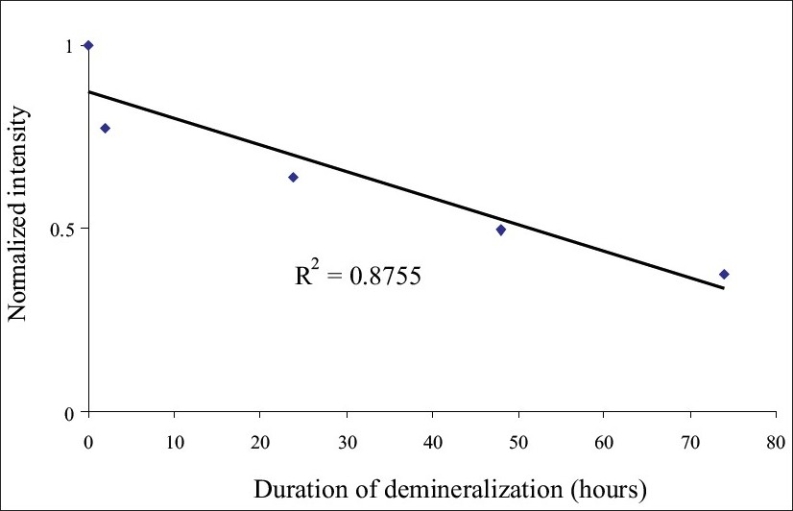
The graph showing the shift in the average normalized backscatter light intensity (at 670 nm), with different intervals of demineralization

**Figure 6:**
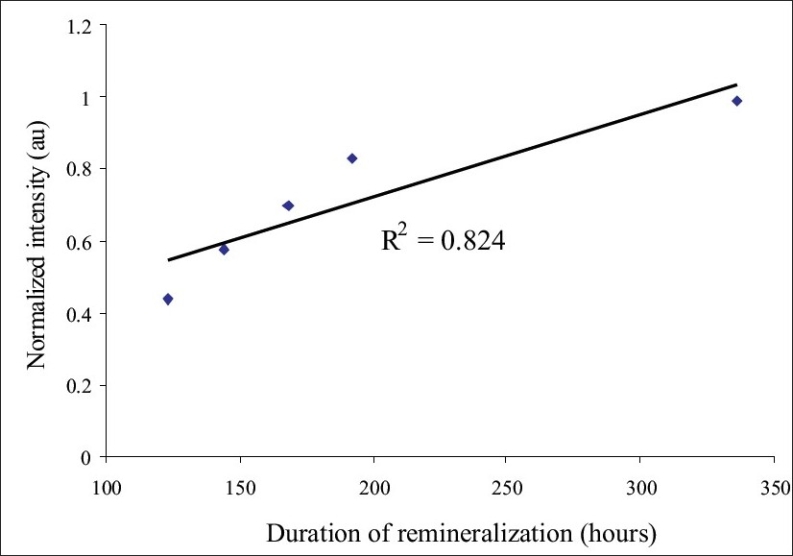
The graph showing the shift in the average normalized backscatter light intensity (at 670 nm), with different intervals of remineralization

*Stage 2: Experiments on tooth specimens:* The spectral pattern obtained from all undemineralized tooth surface was identical for all samples. Figure 5 shows the variation in the average normalized backscatter light intensity (at 670 nm), after different intervals of demineralization. It was observed that enamel demineralization produced a linear decrease in intensity (at 670nm), with increase in the duration of demineralization. Figure 6 shows the average normalized backscatter light intensity after different intervals of remineralization. It was found that remineralization produced an increase in the backscatter light intensity (at 670nm). There was a linear increase in light intensity, with increase in the duration of remineralization.

### Part 2: Examination using polarization microscopy

**Figure 7:**
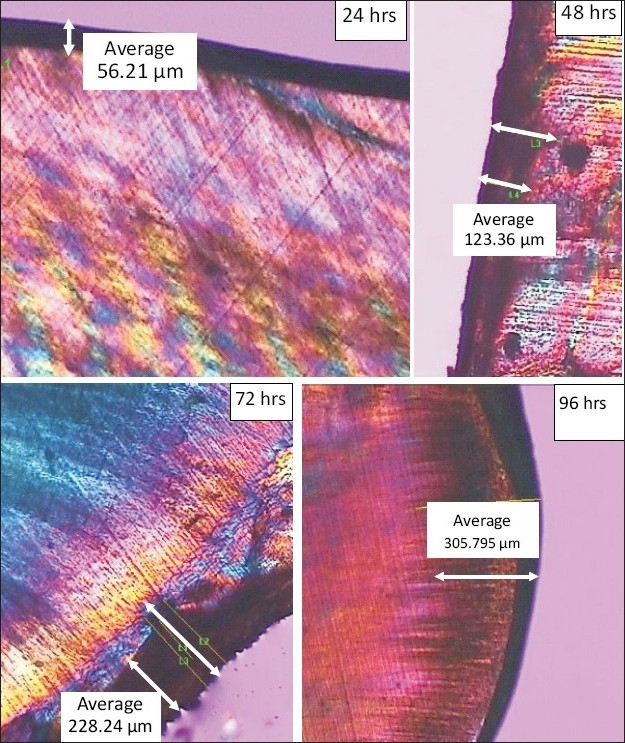
The polarization microscopic images of the demineralized tooth specimens

**Figure 8:**
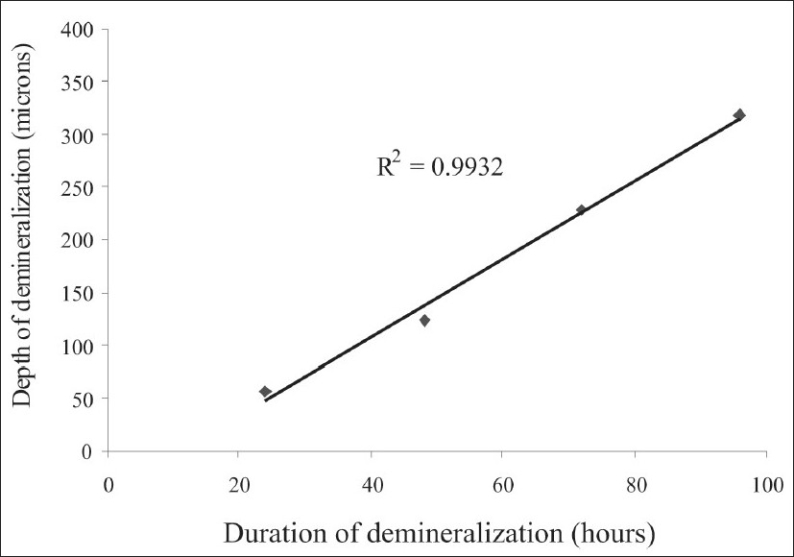
The graph showing the change in the average depth of demineralization as a function of time

Figure 7 shows the polarization microscopic images of the demineralized tooth sections. The average depth of enamel demineralization was 56.21*µ*m, after 24 hours of demineralization; it increased to 318*µ*m after 96 hours of demineralization. There was a linear increase in the depth demineralization in the enamel, with the duration of demineralization [Figure 8]. There was a negative correlation between the increase in the depth of demineralization and the decrease in the backscatter light intensity, with duration of demineralization (*P* = −0.994).

## DISCUSSIONS

Dental caries has been described as a slowly progressing disease process, with potential for remineralization. One of the prerequisite for promoting such remineralization of dental caries is the early detection of the demineralization process and the reproducible, longitudinal monitoring of the lesion. Optical properties are intrinsic properties that characterize a tissue and do not depend on its geometry. Optical methods have played an important role in the study of biological phenomenon. In the past, optical properties such as scattering have been shown to be useful in determining the mineral loss in enamel tissue.[4,21,22,25,26] Although systems based on laser fluorescence show promise, they are relatively expensive, its use is limited to accessible smooth surfaces *in vivo*, and ergonomically compromised for routine use.[19]

The light-tissue interaction between illumination and detection determines the shape and intensity of the resulting spectrum. An optical spectrum represents the collection of constituents in a tissue that interact with light in certain manner. Three types of light-tissue interactions are absorption, scattering, and fluorescence. In scattering, the direction of light travel is changed due to a minute difference in the tissue index of refraction, yet the light intensity remains the same. Absorption reduces the intensity of the light, as photon energy is transferred to the absorbing molecule. Scattering and absorption can occur before light exits the tissue surface, where it can be detected. Fluorescence may occur following absorption, as some of the absorbed energy is released in the form of fluorescent light at the emission wavelength. However, most hard tissues are highly scattering, with the probability of scattering greater than that of absorption, and a small probability of fluorescence occurring following absorption.

Tissue optical spectroscopy is a relatively new area in the field of medical diagnostics, which has the potential to provide automated sensitivity and cost-effective screening for histo-chemical features of tissues.[27] Many tissue molecules are found to possess distinct optical spectra, and optical spectroscopy can be used to record the tissue optical spectra in near real-time and analyzed automatically, without the need for tissue removal. Additionally, with advances in fiber optic technology, remote spectroscopic sensing and monitoring is also possible. This has led to an increasing interest in the development of fiber optic probes for detecting and monitoring diseases, in recent times.[25,28] In this study, the backscattered visible light spectrum from the tooth surface (enamel) was recorded using a bifurcated fiber optic bundle probe connected to a tungsten halogen light source and a high resolution spectrophotometer.

**Figure 9:**
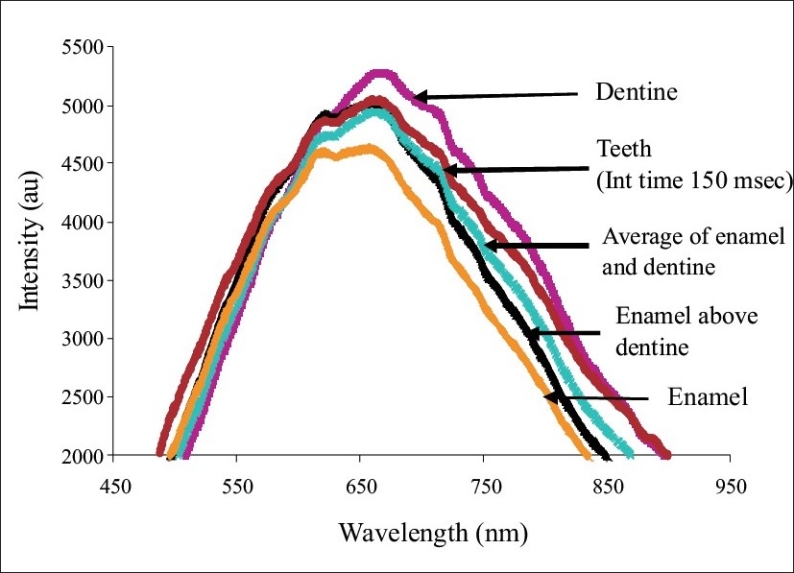
Visible light backscatter spectrum obtained from the enamel, dentine, enamel-dentine combination, average of the enamel and dentine spectra and tooth surface

The experiments in Part 1, conducted on enamel and dentine specimens, showed that enamel and dentine produced distinct backscatter spectra. When the spectrum obtained from the enamel-dentine combination, the spectrum generated from the average of enamel and dentine spectral values, and the spectrum obtained from the sound tooth surface were compared, all of them were different from that of enamel alone and showed major characteristics of dentine. This indicated that the backscattered light spectrum obtained from the sound tooth at the integration time utilized in this study was predominantly from the dentine [Figure 9]. It was observed from the experiments in Part 2 that demineralization produced a linear decrease in the backscatter light intensity from the tooth surface. A good negative correlation was also observed between the decrease in the backscatter light intensity determined using FOBSS and the depth of demineralization measured using polarization microscopy. Further, when FOBSS was tested on demineralized teeth, the specimens subjected to remineralization, there was also an obvious increase in the backscatter light intensity with duration of remineralization. The spectroscopic system used in this study permitted controlling the integration time. This enabled the operator to regulate the number of photons integrated to generate the spectrum of the specimen. In this experiment, an integration time of 150 msec was used to record backscatter light spectra from teeth surfaces (Part 1, Stage 2). This integration time was chosen so that the initial peak from non-demineralized enamel surface resembled the average spectra of enamel and dentine or the spectra obtained from enamel specimen placed above the dentine. This approach will allow the operator to circumvent errors due to variation of enamel thickness at different locations of the crown.

**Figure 10:**
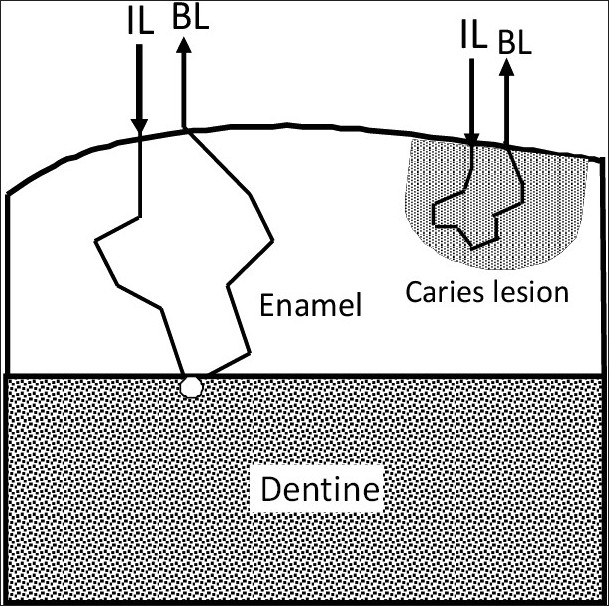
Schematic diagram showing the scattering process in sound and demineralized tooth enamel

Having observed a close relationship between the variations in the fiber optic backscatter light intensity with demineralization and remineralization, it is of significant importance to determine why such changes occur. The Part 1 experiments on enamel and dentine samples vividly showed that the backscatter visible light spectrum obtained from the tooth surface is predominantly from the dentine. This is because the normal enamel has a prism structure with waveguide properties, and when a tooth surface is irradiated, the light will penetrate deep into the enamel.[29] Subsequent to demineralization, the light scatters and alters its path of light in the lesion. Since caries and/or demineralization in the enamel scatters and absorbs more light than the surrounding healthy tissue, the light scattering in the lesion acts as (1) a barrier for the excitation light to interact with the underlying dentine, and (2) as a barrier for the light from the dentine to reach the surface [Figure 10].[30] This study highlights the potential of FOBSS for the chairside detection and monitoring of demineralization and remineralization in tooth. The FOBSS system can not only detect early enamel demineralization and remineralization but also provide useful quantitative information that can be used to monitor changes in a tooth over a period of time or can be used to make comparisons between teeth. The FOBSS system can differentiate enamel and dentine and will enable the operator to circumvent errors due to variation in the thickness of enamel, at different locations of the crown.

## CONCLUSIONS

In this study, a direct fiber optic sensor system that monitors changes in the backscatter light is applied to monitor the demineralization and remineralization induced changes in the enamel surface. In this system, a fiber optic bundle probe is utilized, in which one fiber serves to illuminate the tooth surface while some fibers serve to acquire the backscattered light from the tooth. A broad spectrum visible light source is used for illumination, while a high resolution spectrophotometer is used to acquire the complete spectrum of backscattered light. This study highlights the potential of fiber optic backscatter sensor for the chairside detection and monitoring of demineralization and remineralization in enamel. This sensor can not only detect early enamel demineralization and remineralization but also provide useful quantitative information that can be used to monitor changes in a tooth over a period of time or can be used to make comparisons between teeth. Further, this approach will enable the operator to circumvent errors due to variation in the thickness of enamel at different locations of the crown.
